# Impact of social determinants of health on improving the LACE index for 30-day unplanned readmission prediction

**DOI:** 10.1093/jamiaopen/ooac046

**Published:** 2022-06-10

**Authors:** Anas Belouali, Haibin Bai, Kanimozhi Raja, Star Liu, Xiyu Ding, Hadi Kharrazi

**Affiliations:** Biomedical Informatics and Data Science (BIDS), Division of General Internal Medicine, Johns Hopkins University School of Medicine, Baltimore, Maryland, USA; Biomedical Informatics and Data Science (BIDS), Division of General Internal Medicine, Johns Hopkins University School of Medicine, Baltimore, Maryland, USA; Biomedical Informatics and Data Science (BIDS), Division of General Internal Medicine, Johns Hopkins University School of Medicine, Baltimore, Maryland, USA; Biomedical Informatics and Data Science (BIDS), Division of General Internal Medicine, Johns Hopkins University School of Medicine, Baltimore, Maryland, USA; Biomedical Informatics and Data Science (BIDS), Division of General Internal Medicine, Johns Hopkins University School of Medicine, Baltimore, Maryland, USA; Biomedical Informatics and Data Science (BIDS), Division of General Internal Medicine, Johns Hopkins University School of Medicine, Baltimore, Maryland, USA; Department of Health Policy and Management, Johns Hopkins Bloomberg School of Public Health, Baltimore, Maryland, USA

**Keywords:** social determinants of health, unplanned readmissions, LACE, predictive modeling

## Abstract

**Objective:**

Early and accurate prediction of patients at risk of readmission is key to reducing costs and improving outcomes. LACE is a widely used score to predict 30-day readmissions. We examine whether adding social determinants of health (SDOH) to LACE can improve its predictive performance.

**Methods:**

This is a retrospective study that included all inpatient encounters in the state of Maryland in 2019. We constructed predictive models by fitting Logistic Regression (LR) on LACE and different sets of SDOH predictors. We used the area under the curve (AUC) to evaluate discrimination and SHapley Additive exPlanations values to assess feature importance.

**Results:**

Our study population included 316 558 patients of whom 35 431 (11.19%) patients were readmitted after 30 days. Readmitted patients had more challenges with individual-level SDOH and were more likely to reside in communities with poor SDOH conditions. Adding a combination of individual and community-level SDOH improved LACE performance from AUC = 0.698 (95% CI [0.695–0.7]; ref) to AUC = 0.708 (95% CI [0.705–0.71]; *P* < .001). The increase in AUC was highest in black patients (+1.6), patients aged 65 years or older (+1.4), and male patients (+1.4).

**Discussion:**

We demonstrated the value of SDOH in improving the LACE index. Further, the additional predictive value of SDOH on readmission risk varies by subpopulations. Vulnerable populations like black patients and the elderly are likely to benefit more from the inclusion of SDOH in readmission prediction.

**Conclusion:**

These findings provide potential SDOH factors that health systems and policymakers can target to reduce overall readmissions.

## INTRODUCTION

Unplanned 30-day readmissions are a significant financial burden on the US health care system. As reported by the Center for Medicare and Medicaid Services (CMS), 2 million patients are readmitted annually in the United States, costing Medicare $26 billion. It is also estimated that $17 billion of that cost comes from potentially avoidable readmissions.^1^ Reducing avoidable hospital readmissions has been a key focus of health policies and programs, such as the CMS Hospital Readmission Reduction Program, as the US health care system moves to value-based care.^2^

The Centers for Disease Control and Prevention (CDC) defines SDOH as conditions in which people are born, grow, live, work, and age.^3^ SDOH at the individual level can be related to a person’s age, ethnic background, marital status, or other social factors. At the community level, SDOH are conditions in places where people live that affect their health and quality of life. The community social factors are often grouped into domains related to economic stability, education access, health care access, neighborhood and built environment, and social and community context.^3^ A growing body of evidence indicates that both individual- and community-level SDOH factors impact a patient’s readmission risk.^4^^,^^5^ Consequently, incorporating SDOH into 30-day readmission prediction tools or interventions can potentially improve discharge planning and mitigate readmission rates.^5^^,^^6^

Early and accurate prediction of patients at risk of readmission is key to reducing costs and improving health outcomes. An increasing number of predictive models have been developed to identify patients at high risk of 30-day readmissions.^7^ With advances in predictive modeling techniques, new models tend to be complex and prioritize discrimination over interpretability.^7^^,^^8^ The use of models that leverage simple algorithms can offer transparency in terms of feature interpretation, which is beneficial in clinical settings.^9^ LACE index is a simple and widely validated risk score that is used to predict 30-day readmissions.^10^ Nonetheless, LACE uses exclusively clinical factors (length of stay, acuity, comorbidities, and emergency visits in the last 6 months) and does not account for known contributors to readmission risk such as SDOH.

Previous literature reported an increase in the performance of LACE when incorporating additional variables from electronic health records and other sources.^11^^,^^12^ An international study showed that incorporating a combination of demographics, markers of hospitalization severity, past healthcare utilization, and SDOH increases performance of readmission prediction.^11^ The study only included 2 SDOH variables, namely, the requirement of financial assistance and admission to a subsidized hospital ward. It is unclear how much these SDOH variables alone contributed to improving the LACE model. In another study,^12^ the authors trained an artificial neural network using a large number of electronic health records and census tract SDOH variables. While the artificial neural network model outperformed LACE, no attempt was made to augment LACE itself. Another study on an urban safety-net population found that augmenting LACE with the Area Deprivation Index and individual-level SDOH such as homelessness, learning barriers, and language preferences, increased its performance.^13^ While these studies were successful in improving LACE, they provided limited details on the impact of SDOH variables alone and the importance of these factors in the augmented models. Previous work also did not compare the impact of individual-level SDOH and community-level SDOH on LACE.

In this study, our first objective was to assess the impact and importance of SDOH variables in improving the prediction performance of LACE for unplanned 30-day readmission. We examine the value of including different sets of SDOH predictors, namely, individual-level SDOH, community-level SDOH, and a combination of both. In our second objective, we investigated whether the added predictive value of SDOH varies by demographic subpopulations. To assess this hypothesis, we compare the performance of the models on 8 different demographic subgroups stratified by age, sex, and race.

## MATERIALS AND METHODS

### Study setting and population

This is a retrospective analysis of Maryland’s Healthcare Cost and Utilization Project (HCUP) data.^14^ We used the State Inpatient Database (SID) and the State Emergency Department Database (SEDD) from HCUP. Community-level SDOH variables were extracted from the County Health Ranking (CHR) database,^15^ which includes variables related to health outcomes and social and economic factors for each county in the United States.

**Figure 1.**
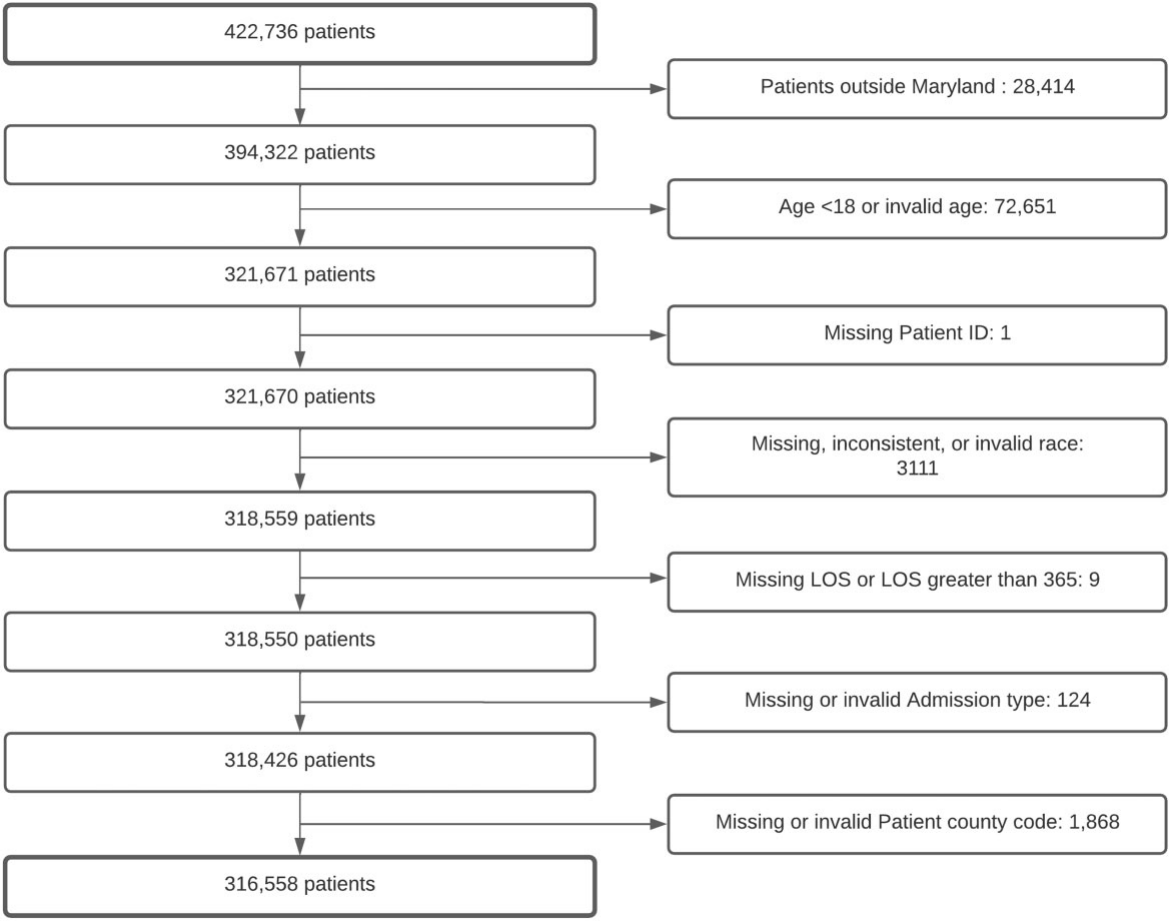
Study population selection process.

Our study population included all inpatient encounters in Maryland from January to December 2019. The initial denominator of the study population included 422 736 patients. We dropped 72 621 patients aged less than 18 years old, because the original LACE index was developed for the adult population. We also excluded patients with residential zip codes outside Maryland; patients with inconsistent race records; invalid or missing admission type or length of stay (LOS); and invalid or missing county codes. In addition, we dropped encounters with missing patient ID, age, and race. After applying the inclusion and exclusion criteria, 316 558 participants were retained to conduct the final analysis (Figure 1).

### Predictors and outcome

The primary outcome of interest is 30-day all-cause unplanned readmission. We defined unplanned readmissions as subsequent inpatient admissions that occur within 31 days of the discharge date of an inpatient visit and have an admission type of emergency or urgent. We used the all-cause readmission variable provided in HCUP and updated readmission status based on the admission type of subsequent admissions. The predictors for the study included the LACE index and its components, individual-level SDOH, and community-level SDOH.

The LACE index is a widely validated model that predicts patients at risk for readmission or death within 30 days of discharge. The LACE index incorporates 4 covariates: LOS, admission type, Charlson Comorbidity Index (CCI), and emergency department (ED) visits within the last 6 months. We calculated the LACE score using inpatient and ED data from the SID and SSED databases. We used the LACE Index weights from the original study^10^ to compute a LACE score for each patient.

For individual-level SDOH, we used a list of international classification of diseases (ICD-10) codes related to social risk factors to identify different SDOH factors that may influence a patient’s readmission status (Supplementary Table S4). The ICD-10 list was adapted from the compendium of SDOH codes compiled by the Social Interventions Research and Evaluation Network (SIREN).^16^ These variables include access to healthcare, homelessness, housing, stress, utilities, social connections and isolation, incarceration, clothing, and marital status.

For community-level SDOH, we used county-level variables from the CHR database. The CHR variables cover different health factor domains. We selected a subset of measures based on completeness and a literature review of community factors that were previously studied for their association with readmission rates.^4^ Community-level variables were highly correlated. We assessed multicollinearity using the Pearson correlation coefficient and dropped highly correlated variables (absolute *r* value larger than 0.7). We also assessed variance inflation factor as an indicator of multicollinearity and required variables used in the model to have a variance inflation factor less than 10.

### Model development and statistical analysis

We investigated the differences in demographics, individual SDOH, and community SDOH between patients with and without 30-day readmissions. We also compared readmissions by LOS and CCI. We used the chi-square test for categorical variables and *t* test for continuous variables to compare readmitted and nonreadmitted patients. We define statistical significance as *P-*value <.05.

We randomly split the dataset into 50% training and 50% testing sets. Given our large dataset, we performed an equal split to increase the size of our testing set. This can produce performance estimates that generalize better to unseen data. We constructed predictive models for the 30-day readmission outcome using Logistic Regression (LR) on different sets of predictors. First, we developed a base model using the LACE index components as predictors. Second, we added individual-level SDOH variables to the base model. Third, we developed a model using community-level SDOH variables and the LACE components. For community-level SDOH variables, we applied Lasso regression for feature selection on the training set and retained a subset of top predictors. Finally, we built a model using individual SDOH, the subset of community-level SDOH, and the LACE components. 

For model building and selection, we performed 3-fold cross-validation (CV) on the training set. The data partitioning for CV was stratified to account for class imbalance. This means that each fold of the CV split had the same percentage of 30-day readmissions as the original dataset. During each iteration of the CV, we kept 1 partition for testing and used the 2 remaining folds to search for the optimal model. We used a grid search to tune LR across a range of hyperparameter settings (ie, L1 and L2 penalty, class weights, and the inverse of regularization strength). The final generalization error was estimated by averaging area under the curve (AUC) scores over the held-out fold. The best LR hyperparameters were used to refit the classifiers on the whole training data. We evaluated performance on the 50% test data set and computed AUC, sensitivity, specificity, positive predictive value, negative predictive value, and Brier score. We used the Youden index to identify the optimized prediction threshold to balance sensitivity and specificity. Receiver operating characteristic curves and confusion matrices were used to illustrate the performance of the models. Finally, we used SHapley Additive exPlanations (SHAP) values for feature importance. SHAP is a game-theoretic approach to explain the predictions of machine learning models by computing the contribution of each feature to the predictions made by the model.^17^

To account for within county variations, we conducted a sensitivity analysis. We fit 2 mixed-effects logit models to the training data for both the community-level SDOH and the all-level SDOH models.

All statistical modeling were completed using Python scikit-learn 0.23.0^18^ and statsmodels v0.13.2.^19^

## RESULTS

### Population characteristics and readmission

Our study population included 316 558 patients of which 35 431 (11.19%) patients were readmitted after 30 days (Table 1). The mean age of the population was 55.9 (SD = 21.1) years, 61.6% were women, 43.2% were married, 54.7% were white, and 33.0% were black. The mean LOS was 4.49 (SD = 6.55) days, 36.7% had no comorbidities (CCI = 0), and 7.4% had a CCI of 5 or higher. The average LACE score was 7.17 (SD = 3.94) and 28.2% of patients were in the higher readmission LACE risk group (LACE ≥ 10).

**Table 1.** Population characteristics by readmission status (*N* = 316 558)

Characteristic		Total	No readmission	Readmission^a^	*P*-value^b^
		(*N* = 316 558)	(*N* = 281 127)	(*N* = 35 431)	
Sex	Male	121 444 (38.4%)	104 540 (37.2%)	16 904 (47.7%)	<.001
	Female	195 114 (61.6%)	176 587 (62.8%)	18 527 (52.3%)	
Age	Mean (SD)	55.9 (21.1)	55.0 (21.2)	63.1 (18.4)	<.001
Race	White	173 207 (54.7%)	152 349 (54.2%)	20 858 (58.9%)	<.001
	Black	104 410 (33.0%)	92 611 (32.9%)	11 799 (33.3%)	
	Other	38 941 (12.3%)	36 167 (12.9%)	2774 (8.1%)	
Marital status	Married	136 857 (43.2%)	123 220 (43.8%)	13 637 (38.5%)	<.001
	Others	175 360 (55.4%)	153 886 (54.7%)	21 474 (60.6%)	
	Missing	4341 (1.4%)	4021 (1.4%)	320 (0.9%)	
Homeless status	*n* (%)	391 (0.1%)	330 (0.1%)	61 (0.2%)	.007
Access to health care	*n* (%)	708 (0.2%)	504 (0.2%)	204 (0.6%)	<.001
Clothing	*n* (%)	40 (0.0%)	30 (0.0%)	10 (0.0%)	.012
Food	*n* (%)	786 (0.2%)	495 (0.2%)	291 (0.8%)	<.001
Housing	*n* (%)	136 (0.0%)	100 (0.0%)	36 (0.1%)	<.001
Incarceration	*n* (%)	2346 (0.7%)	1750 (0.6%)	596 (1.7%)	<.001
Safety	*n* (%)	1984 (0.6%)	1537 (0.5%)	447 (1.3%)	<.001
social connections	*n* (%)	4958 (1.6%)	3720 (1.3%)	1238 (3.5%)	<.001
Stress	*n* (%)	2906 (0.9%)	2070 (0.7%)	836 (2.4%)	<.001
Utilities	*n* (%)	72 (0.0%)	52 (0.0%)	20 (0.1%)	<.001
Length of stay	Mean (SD)	4.49 (6.55)	4.18 (6.04)	7.00 (9.33)	<.001
Charlson Comorbidity Index	0	116 296 (36.7%)	111 823 (39.8%)	4473 (12.6%)	<.001
	1–2	122 199 (38.6%)	109 257 (38.9%)	12 942 (36.5%)	
	3–4	54 609 (17.3%)	43 827 (15.6%)	10 782 (30.4%)	
	≥5	23 454 (7.4%)	16 220 (5.8%)	7234 (20.4%)	
LACE risk groups	Low (0–4)	94 275 (29.8%)	91 881 (32.7%)	2394 (6.8%)	<.001
	Moderate (5–9)	133 101 (42.0%)	119 662 (42.6%)	13 439 (37.9%)	
	High (≥10)	89 182 (28.2%)	69 584 (24.8%)	19 598 (55.3%)	
LACE Score	Mean (SD)	7.17 (3.94)	6.80 (3.83)	10.0 (3.58)	<.001

a Patients with at least one readmission.

b *P*-value is calculated for readmission vs no-readmission using *t* test for continuous variables and chi-square test for categorical variables.

Unadjusted comparisons in Table 1 show that patients with unplanned 30-day readmissions had a higher LOS of 7.00 (SD = 9.33) compared to nonreadmitted patients 4.18 (SD = 6.04). Males were less likely to be readmitted (47.7%) compared to females (52.3%). Readmitted patients were older with a mean age of 63.1 (SD = 18.4) years compared to 55.0 (SD = 21.2) years for nonreadmitted patients. Compared to patients without readmission, patients with unplanned readmissions were more likely to be white (58.9% vs 54.2%), and less likely to be married (38.5% vs 43.9%). Patients with a CCI of 5 or higher were more likely to be readmitted (20.4% vs 5.8%).

Overall, patients with unplanned 30-day readmissions had poor individual-level SDOH conditions compared to patients without readmissions. A higher proportion of readmitted patients had challenges with accessing healthcare (0.6% vs 0.2%); challenges with food security (0.8% vs 0.2%); problems related to legal circumstances or incarceration (1.7% vs 0.6%); challenges related to physical or emotional safety (1.3% vs 0.5%); isolation and lack of social connections (3.5% vs 1.3%); and challenges with stress (2.4% vs 0.7%). All individual-level SDOH differences between the 2 groups were statistically significant. Supplementary Table S1 shows the prevalence of individual-level SDOH by demographic subgroups.

Table 2 presents unadjusted comparisons between readmitted and nonreadmitted patient groups. Patients with 30-day readmissions were more likely to reside in communities with poor SDOH. Readmitted patients came from counties with a higher proportion of smokers (14.2% vs 13.8%), higher firearm fatalities rate (13.6% vs 12.5%), higher excessive drinking (17.0% vs 16.8%), higher single-parent households rate (36.1% vs 35.7%), more violent crimes (567 per 100k vs 522 per 100k), higher injury deaths (80.8 per 100k vs 75.7 per 100k), higher rate of driving alone to work (74.1% vs 73.8%), and lower (worse) food environment index (8.35 vs 8.37). On the other hand, nonreadmitted patients were more likely to live in counties with a higher average household income (78.6k vs 77.3k), and a higher percentage of uninsured adults (8.28% vs 7.95%) and uninsured children (3.39 vs 3.26).

**Table 2.** County level SDOH characteristics

Domain	Characteristics	Total	No readmission	Readmission^a^	*P*-value^b^
		(*N* = 316 558)	(*N* = 281 127)	(*N* = 35 431)	
Health behaviors	Smokers (%)	13.8 (3.78)	13.8 (3.77)	14.2 (3.80)	<.001
	Alcohol impaired deaths (%)	28.3 (7.02)	28.4 (7.03)	27.5 (6.88)	<.001
	Chlamydia rate (per 100k)	542 (308)	541 (306)	547 (326)	.001
	Obese (%)	30.8 (4.82)	30.8 (4.86)	30.6 (4.44)	<.001
	Food environment index	8.37 (0.962)	8.37 (0.958)	8.35 (0.989)	.002
	Excessive drinking (%)	16.8 (1.68)	16.8 (1.69)	17.0 (1.63)	<.001
	Perc food insecure	11.3 (5.28)	11.2 (5.25)	11.4 (5.55)	<.001
	Motor vehicle mortality rate	9.02 (2.84)	9.03 (2.84)	8.93 (2.78)	<.001
Clinical care	Primary care physicians rate (per 100k)	86.3 (37.0)	86.1 (37.1)	87.9 (36.2)	<.001
	Mental health providers rate (per 100k)	240 (90.1)	239 (89.9)	250 (90.6)	<.001
	Preventable hospital stays rate (per 100k)	4810 (1060)	4800 (1060)	4860 (1070)	<.001
	Uninsured adults (%)	8.24 (2.25)	8.28 (2.28)	7.95 (1.98)	<.001
	Uninsured children (%)	3.38 (0.753)	3.39 (0.762)	3.26 (0.669)	<.001
Social and economic environment	Unemployed (%)	4.35 (1.04)	4.35 (1.05)	4.36 (0.991)	.129
	Residential segregation index				
	Mean (SD)	49.9 (10.6)	49.7 (10.5)	51.1 (10.9)	<.001
	Missing	1175 (0.4%)	1061 (0.4%)	114 (0.3%)	
	Firearm fatalities rate				
	Mean (SD)	12.7 (10.5)	12.5 (10.4)	13.6 (11.2)	<.001
	Missing	1155 (0.4%)	1010 (0.4%)	145 (0.4%)	
	Single parent households (%)	35.7 (13.2)	35.7 (13.1)	36.1 (13.9)	<.001
	Social association rate (per 10k)	9.17 (1.98)	9.17 (1.99)	9.21 (1.87)	<.001
	Violent crime rate (per 100k)	527 (448)	522 (443)	567 (480)	<.001
	Injury death rate (per 100k)	76.2 (32.1)	75.7 (32.0)	80.8 (33.0)	<.001
	Household income ($1k)	78.5 (20.1)	78.6 (20)	77.3 (20.5)	<.001
Physical environment	Average Daily PM2.5	10.2 (0.650)	10.2 (0.651)	10.3 (0.642)	<.001
	Drinking water violations	34 910 (11.0%)	31 019 (11.0%)	3891 (11.0%)	.776
	Severe housing problems (%)	17.0 (3.36)	17.0 (3.35)	16.9 (3.46)	<.001
	Drive alone (%)	73.8 (8.71)	73.8 (8.70)	74.1 (8.82)	<.001
	Long commute drives alone (%)	47.8 (9.05)	47.8 (9.16)	47.2 (8.10)	<.001
Demographics	Age 65 or older (%)	15.1 (2.52)	15.1 (2.54)	15.2 (2.30)	<.001
	Female (%)	51.6 (1.18)	51.6 (1.17)	51.6 (1.25)	.064
	Rural (%)	12.9 (16.2)	12.9 (16.2)	12.8 (16.2)	.153

SD, standard deviation.

a Patients with at least one readmission.

b *P*-value is calculated for readmission vs no-readmission using *t* test for continuous variables and chi-square test for categorical variables.

### Model performance and interpretability

As presented in Supplementary Table S2, we found that building the predictive model on LACE components (ie, CCI, LOS, admission type, and the number of ED visits within the previous 6 months) performed better in our population (AUC = 0.698) as opposed to using LACE as a continuous score (AUC = 0.68) or using the recommended cutoff of 10 (AUC = 0.62). Hence, we considered the LACE components model as our baseline to conduct all comparisons.

**Figure 2.**
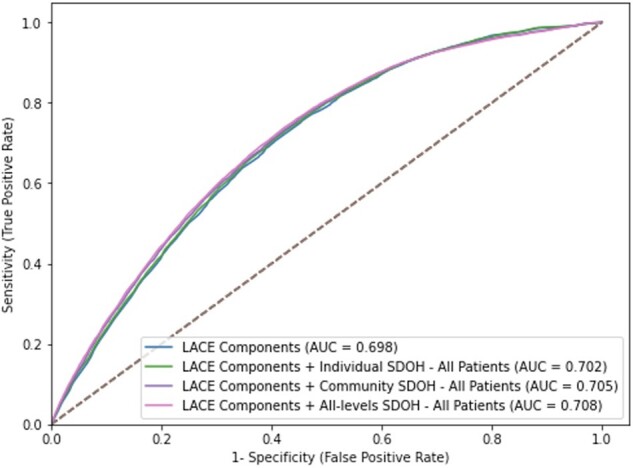
Receiver operating characteristic curves comparing model performances for all patients.

We assessed the performance of the predictive models for 30-day readmission on the test set (Table 3). Receiver operating characteristic curves were generated to compare the different model performances (Figure 2). Confusion matrices for the models are shown in Supplementary Figure S1. The AUC for the LACE base model for the overall population was 0.698 (95% CI [0.695–0.7]; *ref*) and a Brier score equal to 0.22. Adding individual-SDOH to LACE increased slightly the AUC to 0.702 (95% CI [0.698–0.704]; *P *<* *.001) and improved the accuracy (Brier score = 0.09). Adding community level SDOH to the base model had a small but significant improvement in its performance with an AUC = 0.705 (95% CI [0.702–0.707]; *P *<* *.001) but did not have an effect on the Brier score. The combined model with LACE components, individual-level SDOH, and community-level SDOH had the highest improvement compared to the base model with an AUC equal to 0.708 (95% CI [0.705–0.71]; *P *<* *.001) and an improved Brier score equal to 0.09.

**Table 3.** Performance of the predictive models for 30-day readmission on the test set

Model	Brier score	Sens (95% CI)	Spec (95% CI)	PPV (95% CI)	NPV (95% CI)	AUC (95% CI)	*P*-value
LACE components	0.22	0.729(0.724–0.733)	0.569(0.567–0.572)	0.176(0.174–0.178)	0.943(0.942–0.944)	0.698(0.695–0.700)	ref
LACE components+individual SDOH	0.09	0.627(0.622–0.632)	0.665(0.663–0.667)	0.191(0.189–0.193)	0.934(0.933–0.935)	0.702(0.698–0.704)	<.001
LACE components+community SDOH	0.22	0.727(0.722–0.731)	0.582(0.579–0.584)	0.180(0.178–0.182)	0.944(0.943–0.945)	0.705(0.702–0.707)	<.001
LACE components+all-levels SDOH	0.09	0.625(0.619–0.629)	0.676(0.674–0.678)	0.196(0.193–0.198)	0.935(0.933–0.936)	0.708(0.705–0.710)	<.001

Sens, sensitivity; Spec, specificity; PPV, positive predictive value; NPV, negative predictive value; AUC, area under the curve. *P*-value was calculated using Delong test.

The additional predictive effects of SDOH on LACE were assessed in different demographics subgroups (Table 4). Overall, we noticed the same trend of improvement we observed in the general population. Nevertheless, the improvement in discrimination and accuracy varied by cohorts and by the type of SDOH variables added to the base models. Adding individual-level SDOH to LACE improved the Brier score in all cohorts but tended to have a minimal effect on discrimination. The community-level SDOH variables improved discrimination but not the accuracy or calibration. Incorporating both individual and community level SDOH in the LACE models had the highest improvement on discrimination and Brier score in all cohorts. The increase in AUC was highest in black patients (+1.6), patients aged 65 years or older (+1.4), male patients (+1.4), and patients aged 45–64 years (+1.0). However, cohorts of patients aged 18–44 years, white patients, and female patients only had a small increase in AUC. The cohort of patients who are not black or white did not benefit from adding SDOH to LACE.

**Table 4.** Performance of the predictive models for different demographics subgroups on the test set

Subpopulation	Model	Brier score	AUC (95% CI)	*P*-value
White	LACE components	0.22	0.696 (0.692–0.699)	Ref
	LACE+Individual SDOH	0.10	0.700 (0.696–0.703)	<.001
	LACE+Community SDOH	0.22	0.702 (0.699–0.706)	<.001
	LACE+All-levels SDOH	0.10	0.705 (0.702–0.709)	<.001
Black	LACE components	0.23	0.683 (0.677–0.689)	Ref
	LACE+Individual SDOH	0.09	0.687 (0.681–0.693)	<.001
	LACE+Community SDOH	0.22	0.697 (0.691–0.702)	<.001
	LACE+All-levels SDOH	0.09	0.699 (0.693–0.705)	<.001
Other	LACE components	0.20	0.759 (0.750–0.768)	Ref
	LACE+Individual SDOH	0.07	0.761 (0.752–0.768)	.140
	LACE+Community SDOH	0.07	0.762 (0.752–0.771)	.152
	LACE+All-levels SDOH	0.07	0.762 (0.752–0.771)	.151
Female	LACE components	0.22	0.717 (0.712–0.721)	Ref
	LACE+Individual SDOH	0.09	0.720 (0.715–0.725)	<.001
	LACE+Community SDOH	0.08	0.722 (0.718–0.727)	<.001
	LACE+All-levels SDOH	0.08	0.724 (0.720–0.729)	<.001
Male	LACE components	0.23	0.670 (0.665–0.674)	Ref
	LACE+Individual SDOH	0.11	0.673 (0.668–0.677)	<.001
	LACE+Community SDOH	0.23	0.682 (0.677–0.687)	<.001
	LACE+All-levels SDOH	0.11	0.684 (0.680–0.689)	<.001
Age 18–44	LACE components	0.06	0.768 (0.761–0.774)	Ref
	LACE+Individual SDOH	0.06	0.772 (0.766–0.779)	<.001
	LACE+Community SDOH	0.06	0.769 (0.762–0.776)	.107
	LACE+All-levels SDOH	0.06	0.774 (0.767–0.781)	<.001
Age 45–64	LACE components	0.22	0.688 (0.682–0.693)	Ref
	LACE+Individual SDOH	0.11	0.690 (0.685–0.696)	<.001
	LACE+Community SDOH	0.10	0.697 (0.692–0.702)	<.001
	LACE+All-levels SDOH	0.10	0.698 (0.694–0.703)	<.001
Age 65 or older	LACE components	0.23	0.646 (0.641–0.650)	Ref
	LACE+Individual SDOH	0.11	0.648 (0.643–0.653)	<.001
	LACE+Community SDOH	0.23	0.659 (0.654–0.663)	<.001
	LACE+All-levels SDOH	0.11	0.660 (0.655–0.664)	<.001

AUC, area under the curve; *P*-value calculated using Delong test.

**Figure 3.**
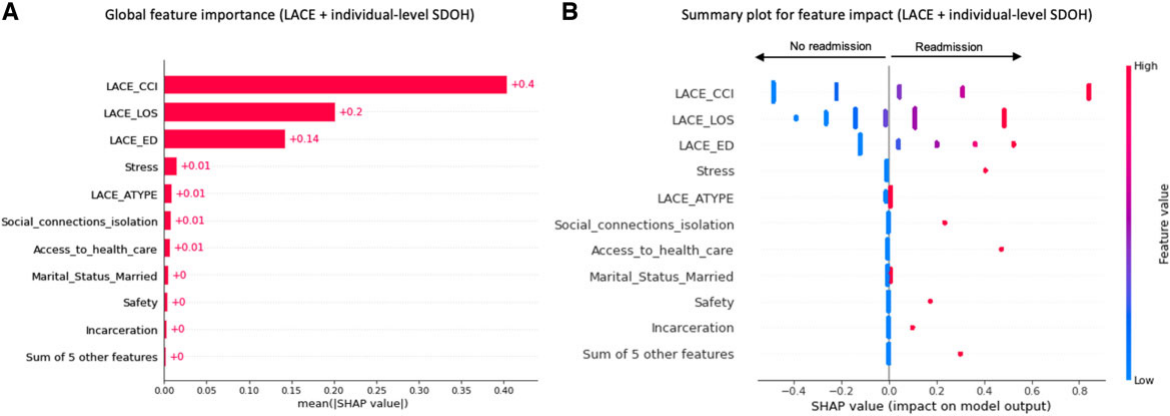
Feature importance of the individual-SDOH augmented LACE model.

**Figure 4.**
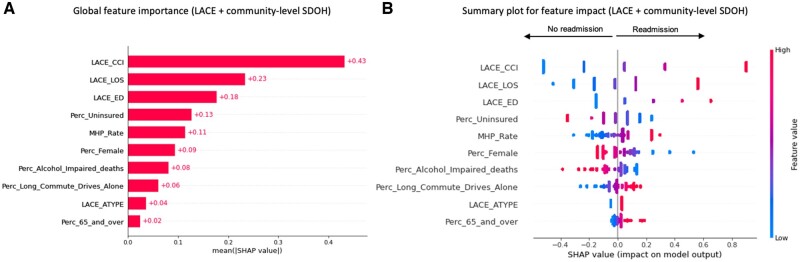
Feature importance of the community-level SDOH augmented LACE model.

**Figure 5.**
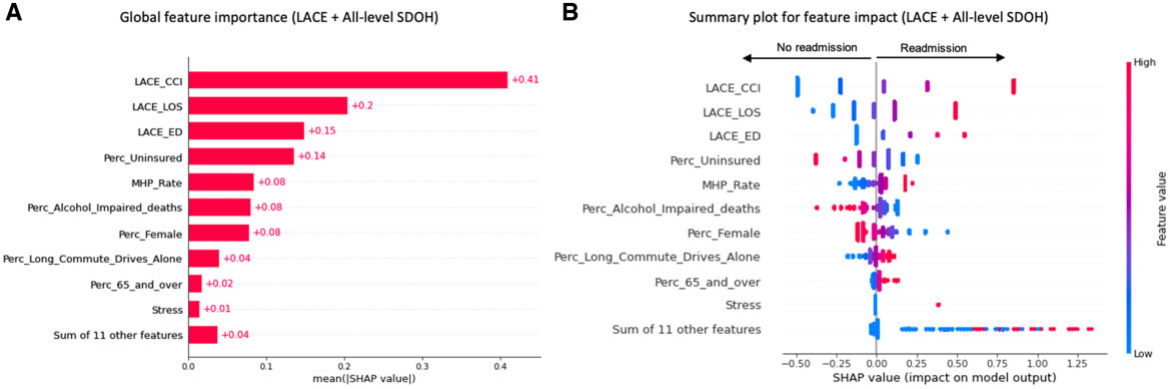
Feature importance of the augmented LACE model with community and individual level SDOH.

We conducted a SHAP values analysis to evaluate the impact of the features in the different SDOH-augmented LACE models for the general population (Figures 3–5). The *x*-axis in the SHAP summary plots denotes the impact of each prediction on the model represented by a dot. Higher SHAP values in the SHAP summary plot (right on the *x*-axis) indicate a higher readmission probability. The *y*-axis represents the predictors in descending order of importance. The gradient color indicates the original value for that variable: red or blue for categorical variables and a spectrum from blue to red for continuous variables. The topmost important individual SDOH predictors associated with higher readmissions for the general population cohort were: stress, isolation or lack of social connections, problems with access to health care, being married, problems with physical or emotional safety, and problems related to legal circumstances or incarceration (Figure 3). The top community SDOH predictors associated with higher readmissions in the community model were: a higher rate of mental health practitioners, a higher percentage of the population who drives alone to work, and a higher percentage of the elderly population (Figure 4). The community SDOH predictors associated with lower readmissions were: a higher percentage of uninsured adults, a higher percentage of female population, a higher percentage of alcohol-impaired deaths (Figure 4). In the LACE plus all-level SDOH model, the top predictors remained the community SDOH predictors we listed previously, followed by stress from individual-level SDOH (Figure 5).

## DISCUSSION

Predicting potentially avoidable readmission has been a key focus of recent research and policy. Social challenges and community factors have been found to impact health outcomes and utilization, including readmission rates.^6^^,^^20^ In this study, we assessed whether incorporating SDOH in the LACE index can improve its prediction performance for unplanned 30-day readmission. Our findings show that the performance of LACE on the general population (AUC = 0.698, *ref*) can be marginally improved by adding individual-level SDOH, community-level SDOH, or a combination of both (AUC = 0.708, *P *<* *.001). Additionally, we found that SDOH improved LACE performance in certain demographic subgroups more than others. Notably, subpopulations that benefited most from adding SDOH are black patients, patients 65 or older, and male patients.

Our results indicate a small but significant improvement in AUC for the general population when adding SDOH to LACE. While the increase of 1% in discrimination may appear negligible, it does translate to the correct classification of an extra 3166 patients in our population. For a calibrated model, this can lead to predicting an additional 354 readmitted patients (ie, 11.19% of 3166 patients). Assuming an average readmission cost of $15 200 per patient,^21^ incorporating SDOH in common readmission models may lead to saving approximately $5.4 Million annually in avoidable costs in the state of Maryland alone.^22^

Our results also illustrate that the additional predictive effects of social determinants on 30-day readmission risk vary by subpopulations. The LACE models built for black, 65 or older, and male cohorts benefited most from adding SDOH. On the other hand, white patients, females, and patients aged 18–44 years benefited less. This could be the result of vulnerable groups such as black patients and the elderly being disproportionately affected by social conditions and being less likely to be able to compensate for these factors. Additionally, male patients are at a much higher risk of readmission compared to women.^5^ Our findings are in line with previous work, which found that adding SDOH to LACE for readmission prediction in an urban safety-net population increased its performance by 2% (from an AUC of 0.65 to 0.67).^13^ Similar work on assessing the impact of individual and community-level SDOH on the HOSPITAL score reported an improved prediction of 30-day readmission for vulnerable patient subgroups including 65 or older, Medicaid, and obese patients.^22^

Our second objective was to identify the top contributing SDOH variables to the improvement of LACE. We conducted a SHAP values analysis to evaluate the impact of the features in the different SDOH-augmented LACE models for the general population. The top 3 individual-level SDOH features that contributed to the LACE plus individual-level SDOH model were variables related to challenges with stress, social connections and isolation, and access to healthcare. All 3 variables have been previously linked to 30-day readmissions as strong predictors.^23–25^

The SHAP summary plot of the community-SDOH LACE model indicates that patients had a higher probability of nonreadmission at 30 days if they lived in communities with a higher percentage of uninsured adults, a higher percentage of females, and a higher percentage of alcohol-impaired driving deaths. On the other hand, patients had a higher probability of readmission if they resided in communities with a higher rate of mental health practitioners, a higher percentage of 65 or older population, and a higher percentage of long commute-drive to work. In fact, previous work has identified the community’s age characteristics, the percentage of workers who have a long commute-drive to work, and the percentage of alcohol-impaired driving deaths, among the 19 most important community variables that predict readmission rates.^4^

In the combined model, the LACE admission type variable was not part of the top 10 predictors. It is possible that access to ED is correlated with community factors. The ranking of the top community-SDOH features in the combined model were similar to the community-level model. The community-SDOH features also had a larger impact on the prediction compared to individual-level SDOH. Only stress from individual-level SDOH was among the top 10 most impactful features. This is possibly due to the lack of collection of individual SDOH data at the point of care.^26^^,^^27^ It is possible that the collection of more comprehensive individual SDOH level data can help improve LACE further.

This study has some limitations. First, this study used data from the state of Maryland only. It is likely that we did not capture readmissions of patients who were readmitted to hospitals in neighboring states, thus leading to an underrepresentation of the true readmission rate. Second, individual-level SDOH data is underreported in hospital discharge data and is likely to be missing for a large proportion of patients. Hence, the top important individual-level SDOH predictors may be different if SDOH were to be captured for all patients. Third, a county is a large geographical area and may not capture more granular SDOH information at the neighborhood level which may be more relevant to readmission. Lastly, the hierarchical nature of the county and individual-level variables may limit the interpretation of the top features for the combined modeling. To address this limitation, we fit a mixed effect logit model to account for group-level variations (ie, county) which showed similar trends of model improvement when adding SDOH (Supplementary Table S3). Future work may focus on investigating the interaction between the different levels of SDOH and incorporating SDOH from other sources at the census tract level or at the neighborhood level. Further research may also investigate other subpopulations including Medicaid patients and conditions with the highest readmission rates (eg, congestive heart failure).

## CONCLUSION

In this study, we demonstrated the value of SDOH in improving the LACE index, a widely used tool to predict the risk of 30-day readmission. We also showed that the additional predictive effects of SDOH on 30-day readmission risk vary by subpopulations. Vulnerable populations like black patients and patients 65 or older are likely to benefit more from the inclusion of SDOH in readmission prediction. We also conducted an examination of the top predictors which can be investigated further in future studies. These findings provide potential SDOH challenges that health systems and policymakers can address to reduce overall hospital readmissions. Future work may examine the collection of SDOH during hospital admission to inform readmission prediction models at discharge.

## FUNDING

This research received no specific grant from any funding agency in the public, commercial, or not-for-profit sectors.

## AUTHOR CONTRIBUTIONS

AB drafted the final manuscript and conducted the modeling. HB contributed to the writing and analysis. KR, SL, XD, and HK contributed to the conception of the analysis and reviewed the results. All authors edited and provided feedback on all versions of the manuscript.

## SUPPLEMENTARY MATERIAL

Supplementary material is available at *JAMIA Open* online.

## CONFLICT OF INTEREST STATEMENT

None declared.

## DATA AVAILABILITY

The data used in this study were provided by the Agency for Healthcare Research and Quality (AHRQ) under license and can be purchased through https://www.hcup-us.ahrq.gov. The derived data underlying this article will be shared on reasonable request to the corresponding author with permission of AHRQ.

## Supplementary Material

ooac046_Supplementary_Data
