# Supplementary material for: Impact of social determinants of health on improving the LACE index for 30-day unplanned readmission prediction
Source: JAMIA Open. 2022 Jun 10;5(2):ooac046. doi: 10.1093/jamiaopen/ooac046 (PMC9185729; doi:10.1093/jamiaopen/ooac046)
Supplement: ooac046_Supplementary_Data [file ooac046_supplementary_data.docx]

**Impact of Social Determinants of Health on Improving the LACE Index for 30-Day Unplanned Readmission Prediction**

Anas Belouali, MEng, MS^1^, Haibin Bai, MS^1^, Kanimozhi Raja, MD, MS^1^, Star Liu, BS^1^, Xiyu Ding, MS^1^,

Hadi Kharrazi, MD, PhD^1,2^

*^1^Johns Hopkins University School of Medicine, Baltimore, MD, USA*

*^2^Johns Hopkins Bloomberg School of Public Health, Baltimore, MD, USA*

# **Supplementary material**

**
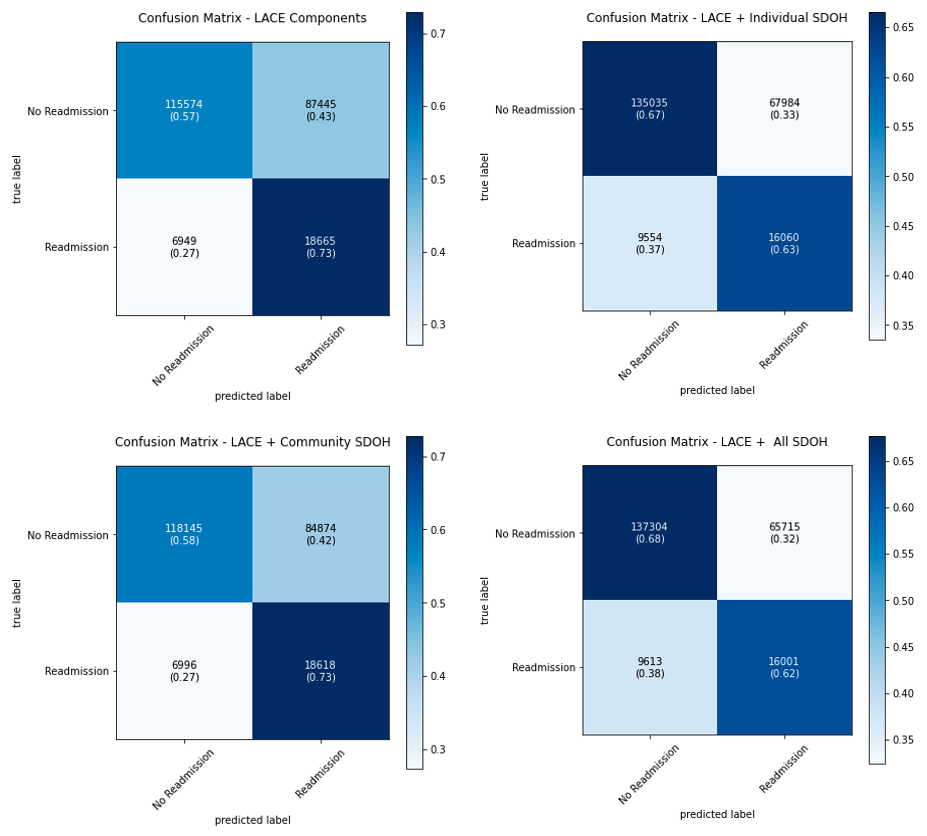
**

**Figure S1.** Confusion matrices for the prediction of different models

| **Table S1.** Individual-level SDOH prevalence in HCUP data by demographics subgroups | | | | | | | | |
| --- | --- | --- | --- | --- | --- | --- | --- | --- |
| **SDOH** | **Race** | | | **Sex** | | **Age** | | |
|  | **White**  **(N=173,207)** | **Black**  **(N=104,410)** | **Other**  **(N=38,941)** | **Male**  **(N=121,444)** | **Female**  **(N=195,114)** | **18-44**  **(N=108,578)** | **45-64**  **(N=83,233)** | **>=65**  **(N=124,747)** |
| **Homeless Status** | 207 (0.1%) | 155 (0.1%) | 29 (0.1%) | 282 (0.2%) | 109 (0.1%) | 200 (0.2%) | 167 (0.2%) | 24 (0.0%) |
| **Access to Health Care** | 388 (0.2%) | 258 (0.2%) | 62 (0.2%) | 348 (0.3%) | 360 (0.2%) | 245 (0.2%) | 301 (0.4%) | 162 (0.1%) |
| **Clothing** | 19 (0.0%) | 19 (0.0%) | 2 (0.0%) | 16 (0.0%) | 24 (0.0%) | 17 (0.0%) | 14 (0.0%) | 9 (0.0%) |
| **Food** | 380 (0.2%) | 377 (0.4%) | 29 (0.1%) | 387 (0.3%) | 399 (0.2%) | 101 (0.1%) | 233 (0.3%) | 452 (0.4%) |
| **Housing** | 66 (0.0%) | 65 (0.1%) | 5 (0.0%) | 65 (0.1%) | 71 (0.0%) | 40 (0.0%) | 58 (0.1%) | 38 (0.0%) |
| **Incarceration** | 1271 (0.7%) | 888 (0.9%) | 187 (0.5%) | 1,430 (1.2%) | 916 (0.5%) | 1,340 (1.2%) | 725 (0.9%) | 281 (0.2%) |
| **Safety** | 986 (0.6%) | 743 (0.7%) | 255 (0.7%) | 393 (0.3%) | 1,591 (0.8%) | 1,275 (1.2%) | 536 (0.6%) | 173 (0.1%) |
| **Social Connections** | 3167 (1.8%) | 1,412 (1.4%) | 379 (1.0%) | 2,218 (1.8%) | 2,740 (1.4%) | 1,713 (1.6%) | 1,357 (1.6%) | 1,888 (1.5%) |
| **Stress** | 1678 (1.0%) | 1,002 (1.0%) | 226 (0.6%) | 1,483 (1.2%) | 1,423 (0.7%) | 1,192 (1.1%) | 986 (1.2%) | 728 (0.6%) |
| **Utilities** | 48 (0.0%) | 20 (0.0%) | 4 (0.0%) | 33 (0.0%) | 39 (0.0%) | 13 (0.0%) | 31 (0.0%) | 28 (0.0%) |

Table S1 shows that patient-level SDOH are not collected systematically in patient discharge data. Nonetheless, some differences are still noticeable between patient subgroups.

**Table S2.** Comparison of the performance of the different LACE-based models

| **Model** | **Brier Score** | **AUC** |
| --- | --- | --- |
| LACE>=10 | 0.348 | 0.624 |
| LACE Score | 0.226 | 0.680 |
| LACE Components | 0.222 | 0.698 |

*Abbreviations: AUC, area under the curve*

Table S2 shows that building a predictive model on LACE components performed better compared to using LACE as a continuous score or using the recommended cutoff of 10.

**Table S3**. Performance of the predictive models for 30-day readmission

| **Model** | **Brier Score** | **Sens** | **Spec** | **PPV** | **NPV** | **AUC** |
| --- | --- | --- | --- | --- | --- | --- |
| LACE Components (LR) | 0.222 | 0.729 | 0.569 | 0.176 | 0.943 | 0.698 |
| LACE Components + Community SDOH - (MixedLM) | 0.266 | 0.743 | 0.560 | 0.176 | 0.945 | 0.700 |
| LACE Components + All level SDOH - (MixedLM) | 0.233 | 0.726 | 0.582 | 0.180 | 0.944 | 0.703 |

*Abbreviations: AUC, area under the curve; LR, Logistic Regression; MixedLM, mixed-effects logit model*

To account for within county variations, we fit two mixed-effects logit models to the training data for both the community-level SDOH and the all-level SDOH models. Table S3 shows the performance of the models on the test set for the general population.

**Table S4.** Lists of individual-level SDOH variables mapped with ICD-10 codes

| **Domain** | **Description** | **ICD-10 Codes** |
| --- | --- | --- |
| **Access to health care** | Difficulty of accessing healthcare service or facilities for some reasons | Z59.7, Z59.8, Z60.8, Z75.3, Z75.9, Z76.8, Z76.89, Z91.1, Z91.120, Z91.8, Z91.89 |
| **Clothing** | Clothing problems including symptoms and signs involving appearance and behavior | R46.8, R46.89 |
| **Food** | Problems or difficulty related to food | E63.9, Z59.4, Z73.8, Z73.89 |
| **Housing** | Problems or difficulty related to housing | Z59.0, Z59.1, Z59.1, Z59.8, Z77.0, Z77.011, Z77.1, Z77.120 |
| **Incarceration** | Problems related to imprisonment, other incarceration, or other legal circumstances | Z60.8, Z63.3, Z63.32, Z63.8, Z65.0, Z65.1, Z65.2, Z65.3 |
| **Safety** | Problems related to various safety issues (e.g., general safety, neighborhood safety, intimate partner violence, etc.) | F40.2 , F40.298, O9A.3 , O9A.31 , O9A.311, O9A.312, O9A.313, O9A.319, O9A.32, O9A.33, O9A.4 , O9A.41 , O9A.411, O9A.412, O9A.413, O9A.419, O9A.42 , O9A.43 , O9A.5 , O9A.51 , O9A.511, O9A.512, O9A.513, O9A.519, O9A.52 , O9A.53 , R45.8 , R45.82 , S09.90, T14.90, S09.9 , T14.9 , T74.0 , T74.02, T74.1 , T74.11, T74.1 , T74.11, T74.1 , T74.12, T74.2, T74.22, T74.2, T74.21, T74.2, T74.21, T74.3, T74.31 , T74.3, T74.31, T74.3, T74.32, T74.9, T74.91, T74.9, T74.91, T74.9, T74.91, T74.9, T74.92, T76.0, T76.02, T76.1, T76.11, T76.1, T76.12, T76.2, T76.21, T76.2, T76.22, T76.3, T76.31, T76.3, T76.32, T76.9, T76.91, X58, Z60.8, Z62.8, Z62.81, Z62.9 , Z63.0 , Z65.4 , Z76.8 , Z76.89 , Z84.8 , Z84.89 , Z91.4 , Z91.41 , Z91.410, Z91.411, Z91.4 , Z91.41, Z91.410, Z91.411, Z91.412, Z91.419, Z91.8 , Z91.89, Z91.8, Z91.89, Z91.8, Z91.89, Z91.8, Z91.89 |
| **Social connections isolation** | Problems related to social skills, living alone and family disruption issues. | R45.8, R45.89, Z60.2, Z60.4, Z60.8, Z63.0, Z63.5, Z63.8, Z63.9, Z65.9, Z73.4, Z91.8, Z91.89 |
| **Stress** | Physical and mental strain related to work, life events, and relaxation. | F43.2, F43.20, F43.9, Z56.3, Z56.6, Z63.7, Z63.79, Z63.8, Z73.2, Z73.3, Z73.8, Z73.89, Z91.8, Z91.89 |
| **Utilities** | Problems with food, water, or other economic circumstances. | Z59.1, Z59.4, Z59.8 |

Table S4 shows a list of ICD-10 codes we used to identify different SDOH factors that may influence a patient’s readmission status. This list was adapted from the compendium of SDOH codes compiled by the Social Interventions Research and Evaluation Network (SIREN).
